# Thermal-stable rotor with enhanced cooling design for efficient electric motor applications

**DOI:** 10.1016/j.ohx.2026.e00770

**Published:** 2026-04-07

**Authors:** Wanwinit Wijittemee, Surasak Noituptim, Sawek Pratummet, Sarun Nakthanom, Boonyang Plangklang

**Affiliations:** a Faculty of Engineering, Rajamangala University of Technology Thanyaburi, Pathum Thani 12110, Thailand; b Sukhothai Thammathirat Open University, Nonthaburi 11000, Thailand

**Keywords:** Induction motor, Poultry farm ventilation fan, Thermal management, Vibration reduction, Motor redesign

## Abstract

This paper presents an open-source redesign of a three‑phase induction motor for poultry farm ventilation fan applications that targets thermal stability, vibration reduction, and higher efficiency. The motor maintains a rated mechanical output of approximately 2 kW, while increases the stator outer diameter (160 → 190  mm) and shortening the axial core length (190 → 110  mm) to conserve D^2^L and shorten the thermal path. The rotor/shaft were shortened accordingly to raise stiffness and the critical speed, lowering vibration. Aluminum die-cast endcaps and a finned AL6063-T5 aluminum housing (a high-conductivity alloy commonly used for motor cooling applications) raised the effective heat-transfer area and conductivity relative to cast iron. Rewinding with a larger wire gauge (0.70  mm × 2) halved phase resistance (18.63  Ω → 8.57  Ω), cutting copper loss. Under full load, input power decreased from 3,420 W to 2,632 W, a reduction of approximately 788 W (23%), and the hottest coil fell by up to 24 °C W (∼16.2%), with rear-section vibration reduced by up to 5.5  mm/s. Complete CAD files, bill of materials, and step‑by‑step build/validation instructions were released under the open-source CERN-OHL-S v2 license (a share-alike hardware license that permits reproduction, modification, and redistribution), enabling replication and adaptation. This work offers a practical template for cost‑effective, thermally robust small induction motors.

## Graphical abstract

**Figure:**
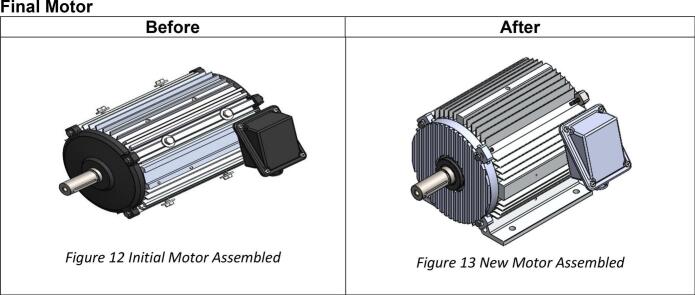

## 1 Introduction

**Specifications table**.

**Table:** 

Hardware name	Thermal-Stable Rotor with Enhanced Cooling for Electric Motor Applications
Subject area	• Engineering and materials science
Hardware type	• Mechanical engineering and materials science
Closest commercial analog	AC induction motor (three-phase, 2 kW, 380 V, 50 Hz) commonly used in poultry farm ventilation fan applications
Open-source license	CERN-OHL-S v2
Cost of hardware	Approx. $131 USD
Source file repository	*Zenodo (open repository) – Concept DOI:*https://doi.org/10.5281/zenodo.17823488

## 2 Hardware in context

**Fig. 1:**
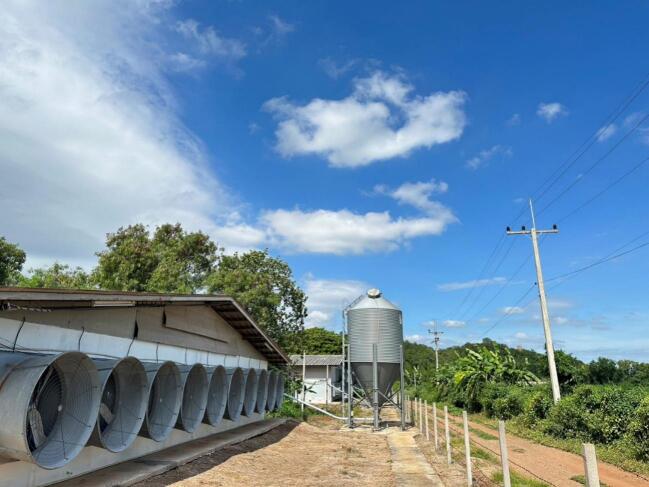
Poultry farm ventilation fan.

This project presents a redesign of a small AC motor for poultry farm ventilation fan applications, focusing on improving efficiency and thermal management through 4 key changes: (1) a stator-rotor with increased outer diameter but reduced axial length, (3) three phase windings with new size and increased turns of winding, (3) a newly designed endcap made from improved material for enhanced cooling and durability and (4) a redesigned housing with more surface area. This approach targets the common problem of overheating and inefficiency in fan motors used in continuous poultry farm ventilation fan operation [1], [2], [3], [4], [5]. The improved structure offers better heat dissipation, reduced power loss, and increased reliability. Fig. 1 illustrates a typical on-site installation, showing multiple farm fan blower units used for continuous airflow, humidity control, and thermal management in a poultry house.

Previous studies have attempted stator slot and winding optimization to improve efficiency and vibration [6], [7], [8], [9], [10], this work introduces a redesign strategy that combines an enlarged stator diameter with a shortened axial length (D^2^L-optimized), aluminum die-cast endcaps, and a finned housing to improve thermal performance. Unlike conventional designs that focus only on winding optimization, our approach integrates mechanical, thermal, and electrical improvements to simultaneously reduce copper loss, enhance heat dissipation, and mitigate vibration [11], [12], [13], [14].

Although increasing stator OD and using aluminum housing have been previously reported, prior studies typically optimize only a single domain. This work proposes a multi-physics redesign framework integrating thermal conduction, mechanical stiffness optimization, and electrical loss reduction, and validates the combined improvement experimentally [14], [15].

## 3 Hardware description

The baseline motor used in this study was originally manufactured at the authors’ industrial motor production facility and has been widely deployed in poultry farm ventilation fan systems. In practical farm environments, these motors operate continuously for extended periods to maintain airflow, humidity control, and thermal regulation inside poultry houses. During long-term field operation under continuous ventilation duty, the original motor exhibited elevated operating temperatures and relatively higher vibration levels, particularly near the rear bearing region. These conditions are commonly observed in fan motors subjected to sustained mechanical loading and limited heat dissipation. Elevated temperature can accelerate insulation aging in stator windings and reduce electrical efficiency, while excessive vibration may increase mechanical stress on bearings and rotating components [16], [17], [18], [19]. These factors can negatively influence the long-term reliability of motors operating in agricultural ventilation systems. To address these limitations, a systematic redesign of the motor structure was carried out in this study while preserving the same fundamental three-phase induction motor architecture. The redesign focuses on improving thermal dissipation, reducing electrical losses, and enhancing mechanical stiffness.

The main structural modifications include:

Increasing the stator outer diameter while reducing axial length following the D^2^L design concept
Redesigning the winding configuration to reduce copper losses [8], [9], [10]
Replacing cast-iron endcaps with aluminum die-cast endcaps for improved thermal conductivity
Introducing a finned aluminum housing to increase convective cooling area
Shortening the rotor and shaft to increase structural stiffness and reduce vibration [16]

The redesigned motor therefore maintains the same functional purpose and application environment as the original unit while incorporating structural modifications intended to improve thermal stability, efficiency, and mechanical reliability. The following subsections describe each design modification in detail.

**Fig. 2:**
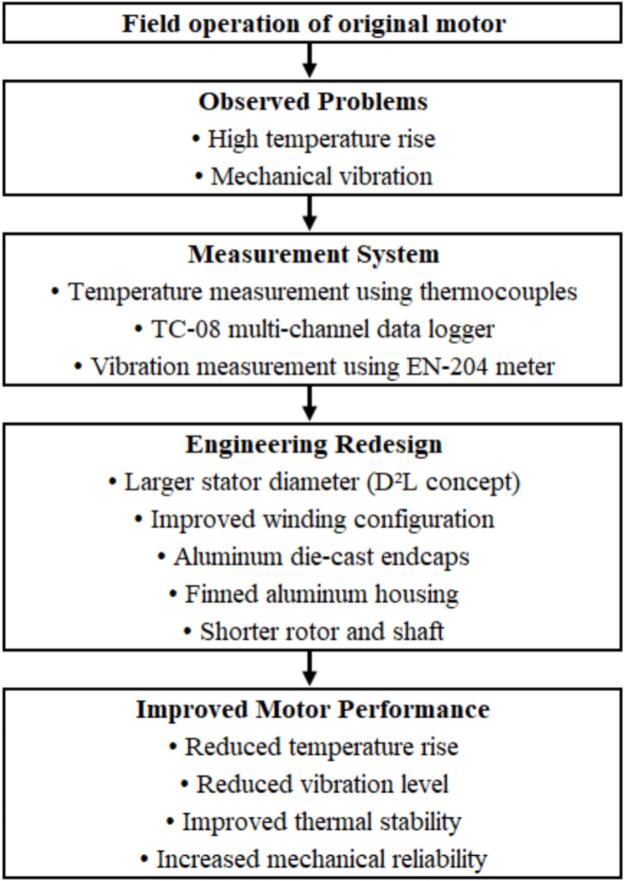
Motor redesign concept and experimental validation process.

The original induction motor used in poultry ventilation systems exhibited elevated operating temperatures and noticeable vibration during continuous field operation. To investigate these issues, temperature and vibration measurements were conducted using thermocouples connected to a multi-channel TC-08 temperature data logger and a handheld EN-204 vibration meter. Based on the measured results, several structural modifications were introduced, including stator geometry adjustment following the D^2^L concept, optimized winding configuration [20], aluminum die-cast endcaps, a finned aluminum housing for improved heat dissipation, and a shortened rotor-shaft structure to increase stiffness. This design improvements contributed to reduced temperature rise and lower vibration levels in the redesigned motor Fig. 2.

### 3.1 Modified stator core

**Fig. 3:**
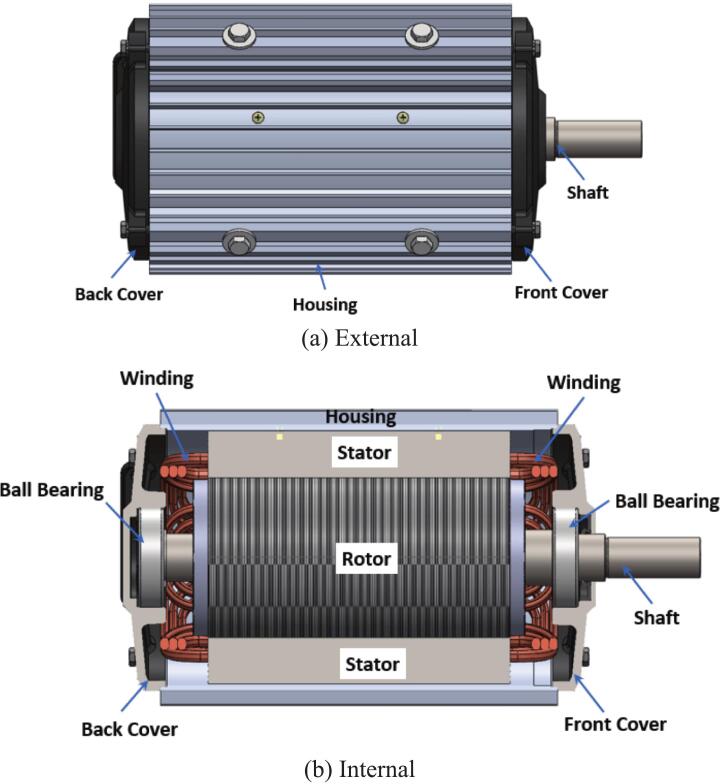
Construction of Initial motor: (a) External view; (b) Internal view.

**Fig. 4:**
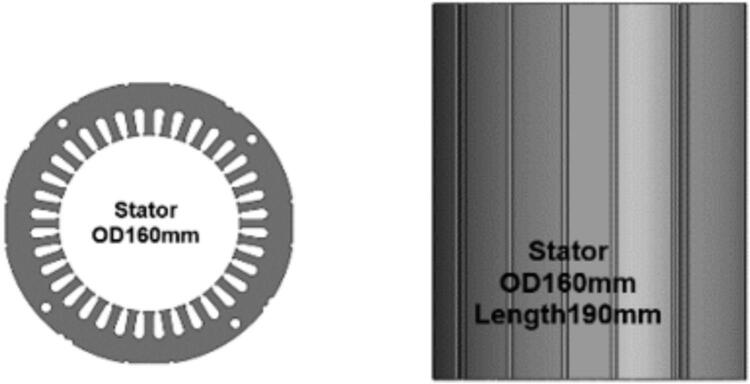
Initial Stator.

The stator core in the initial motor design consisted of laminated 50A1300 electrical steel with an outer diameter of 160 mm and an axial length of 190 mm. As illustrated in Fig. 3, the stator occupied a long cylindrical region surrounding the rotor, which defined the electromagnetic geometry for the original configuration. The redesign focused on modifying this stator geometry while maintaining the same material grade. In the updated design, the outer diameter of the stator was increased to 190 mm, and the axial length was shortened to 110 mm, as shown in Fig. 3 and Fig. 4, respectively. These changes were made to alter the ratio between stator diameter and stack length, shifting from a narrow and long configuration to a wider and shorter stator body.

To establish the relationship between the dimensions of the initial and modified stator, the equivalence expression as shown equation 1.

DA^2^ × LA = DB^2^ × LB (1).

was applied, where DA and LA represent the outer diameter and length of the original stator, and DB and LB represent the corresponding values for the redesign. Substituting the actual values, 160^2^ × 190 = 190^2^ × LB yields a calculated stator length of 134.74 mm for the redesigned configuration. Although the calculated equivalent length was 134.74 mm, the final design selected an axial length of 110 mm to accompany the increased 190 mm diameter. The resulting geometry produces a noticeably shorter stator body while retaining a larger outside diameter to accommodate the increased cross-sectional area.

**Fig. 5:**
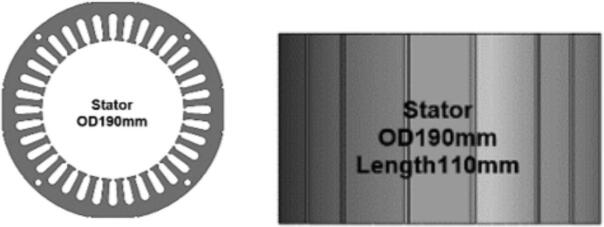
New Stator.

Comparing Fig. 4 and Fig. 5 clearly demonstrates the contrast between the initial and updated stator shapes. The original stator, with the 160 mm diameter and 190 mm stack, forms a taller profile, whereas the redesigned stator exhibits a shortened axial dimension with a larger diameter. In both designs, the laminated steel construction remains unchanged, and the stator is situated concentrically with the rotor in the same manner as in Fig. 3 (b), preserving the general motor layout and assembly method. This redesign presents a straightforward dimensional modification based entirely on adjusting the outer diameter and axial length of the stator while keeping the material selection and overall internal motor arrangement consistent with the initial design.

### 3.2 Modified winding

**Fig. 6:**
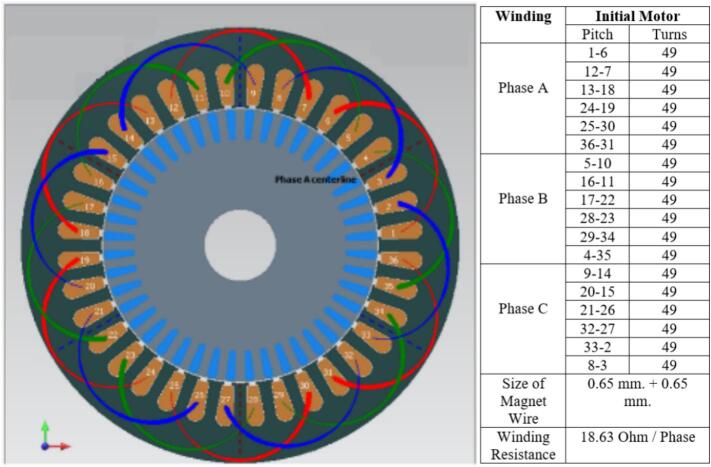
Initial coil design.

The winding configuration [20] was redesigned by modifying both the wire gauge and the number of turns per coil in order to reduce copper losses while maintaining a practical winding space factor. The original winding used copper conductors with a nominal diameter of 0.65 mm, arranged as a three-phase distributed winding with 49 turns per slot. This configuration produced a phase resistance of 18.63 Ω, as listed in the initial coil design table shown adjacent to Fig. 6. The winding layout in Fig. 6 illustrates the original distribution of conductors around the stator slots, where the red, yellow, and blue segments represent the three phases in the coil group. The relatively smaller wire diameter and lower turn count resulted in a moderate coil fill factor but contributed to higher electrical resistance within each phase.

**Fig. 7:**
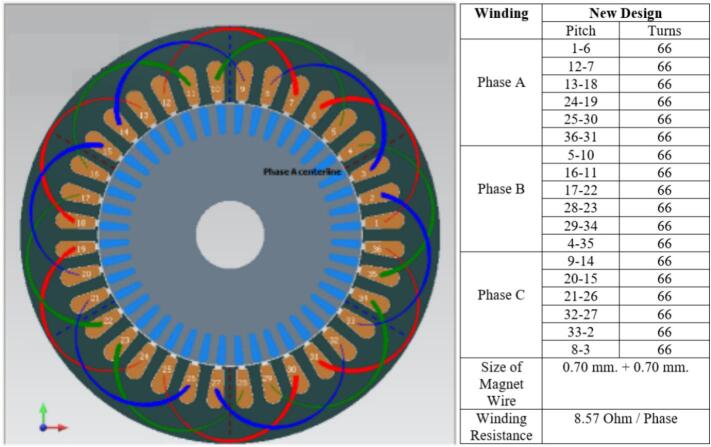
New coil design.

In the revised design, the winding was re-constructed using a slightly larger copper wire with a diameter of 0.70 mm and an increased number of turns, totaling 66 turns per slot. This change in winding parameters led to a reduced phase resistance of 8.57 Ω, as documented in the winding data table presented next to Fig. 7. The corresponding winding layout in Fig. 7 shows a denser distribution of coil segments, indicating the higher copper content per slot in the new configuration. The larger conductor size and greater turn count result in a lower electrical resistance without altering the three-phase coil arrangement or the slot assignment strategy.

Both winding diagrams clearly demonstrate the difference between the original and modified configurations. In Fig. 6, the initial design exhibits fewer conductors per slot, while Fig. 7 contains visibly more conductor paths in the same slot geometry. The coil tables displayed above each figure include the turn values for Phases A, B, and C and summarize the reduction in phase resistance achieved through the updated winding approach. Although the coil distribution pattern remains consistent between the two designs, the increase in copper cross-section, indicated by the shift from 0.65 × 2 to 0.70 × 2 wire size, directly affects the total resistance of the phase circuit. The modification of the winding parameters is further captured by the efficiency expression η= Pout / Pin, where the reduction in copper loss Pcu = 3I^2^R arises from the decreased phase resistance. This relationship is shown in the redesign table accompanying the winding specifications. The new winding arrangement therefore retains the original coil topology and three-phase distribution while altering only the conductor dimensions and turn count, resulting in a lower resistance pathway for current flow. These changes represent a straightforward adjustment to the winding geometry based solely on the content in the table and image comparison between Fig. 6 and Fig. 7, without altering the fundamental electrical configuration of the stator.

Although increasing the number of turns does not directly increase flux density under constant AC voltage excitation, it increases the ampere-turns (NI), which allows the motor to produce the same magnetomotive force with lower phase current. This reduction in current decreases copper losses Pcu = 3I^2^R and improves thermal performance. In addition, the larger conductor cross-section reduces phase resistance, further lowering winding loss while maintaining the original coil topology.

### 3.3 Modified front and rear endcap

The front and rear endcaps in the initial design were manufactured from cast iron, with a total external surface area of approximately 67,826 square millimeters and a representative thermal conductivity of around 50 W/m·K. These endcaps provided the structural mounting interface and rotor alignment but offered only limited heat dissipation due to their relatively low thermal conductivity and minimal external surface geometry [7], [21]. As shown in Fig. 7, the original endcap configuration consists of smooth external faces with few features to promote convective airflow around the assembly.

In the redesigned configuration, both the front and rear endcaps were re-engineered using aluminum die-casting. The new endcaps incorporate integrated bar-fin features distributed across the external face to increase airflow, which enhances cooling performance [21]. Aluminum was selected primarily for its higher thermal conductivity, approximately 160 W/m·K, which represents a 220% improvement over the cast iron values in the initial design, as calculated directly from the thermal conductivity comparison (160 − 50)/50 × 100. The redesigned external geometry also significantly increases the convective surface area to about 122,422 square millimeters, nearly doubling the available external contact area for natural or forced air convection around the motor housing.

**Fig. 8:**
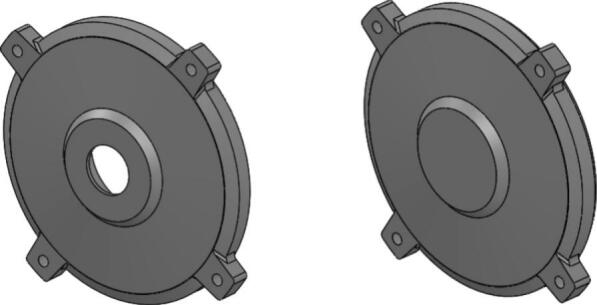
Initial Front and Rear Endcap.

**Fig. 9:**
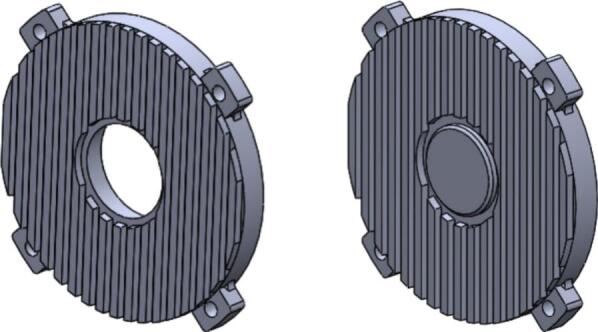
New Front and Rear Endcap.

Fig. 9 shows the new aluminum die-cast endcaps with the integrated bar-fin pattern extending around the circumference of the component. This revised shape provides larger exposed surface segments compared to the smooth cast iron surfaces in Fig. 8, thereby improving airflow contact and heat transfer from the motor interior [7], [21]. While the functional mounting and rotor alignment features remain in similar positions, the external topology has been modified to accommodate cooling efficiency without altering the overall structural role of the endcaps.

The design change therefore focuses on replacing the cast iron endcaps with aluminum die-cast components that offer higher thermal conductivity and expanded surface geometry. This modification is based solely on adjusting the material selection and external shape of the endcaps while maintaining their original mechanical function and interface configuration within the motor assembly Fig. 9.

### 3.4 Modified housing

**Fig. 10:**
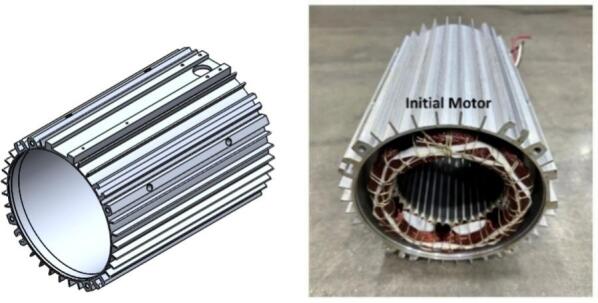
Initial Housing.

**Fig. 11:**
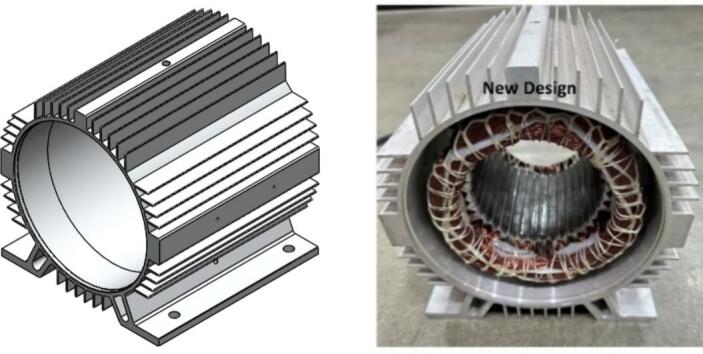
New Housing.

The motor housing in the initial configuration was produced from AL6063-T5 aluminum alloy, with a total surface area of approximately 442,369 square millimeters. This cylindrical structure provided the primary interface for stator mounting and served as the external heat transfer surface during operation. As depicted in Fig. 10, the original housing employed a relatively uniform outer surface without extensive fin features, limiting the available convective area for natural airflow around the motor during poultry farm ventilation fan duty cycles. In the redesigned version, the housing geometry was modified while maintaining the same AL6063-T5 aluminum alloy material. The updated component incorporates a significantly expanded external fin layout, which increases the surface area to approximately 703,811 square millimeters. This represents an estimated surface area improvement of around 59% compared to the original configuration, calculated using the ratio of the new surface value to the original value. The revised housing is shown in Fig. 11, where the denser and deeper fin arrangement on the external surface can be clearly observed. These fins distribute around the full circumference of the housing, offering substantially greater exposure for air contact and heat dissipation.

In addition to surface area changes, the redesigned housing has been dimensioned to accommodate the larger stator diameter used in the updated motor configuration, while preserving the same mounting alignment along the axial direction. This ensures that the modified housing remains compatible with standard assembly fixtures and allows the stator to be positioned precisely without additional structural modifications. The external fin pattern now acts as a combined thermal and structural feature, improving convective heat transfer potential while increasing the overall stiffness of the casing.

The geometry adjustments therefore focus on increasing the external fin density and total surface area of the aluminum housing without altering the fundamental material selection or component function. By comparing Fig. 10 and Fig. 11, the difference between the initial smooth housing and the redesigned finned housing is evident, illustrating how the additional external structure provides greater surface contact area for dissipating heat away from the stator and rotor during operation.

### 3.5 Modified rotor and shaft

The initial rotor lamination consists of 44 equally distributed slots, corresponding to a slot pitch of 8.18°. The slot opening width is 3.3 mm, forming a semi-closed slot profile. The tooth geometry utilizes a dual-radius contour with R46.93 at the top and R34.586 at the bottom, with a fillet radius of R1.595 at the root to reduce stress concentration. This shape supports lower cogging torque and smoother flux distribution while maintaining manufacturability for stamping production.

The new rotor lamination consists of 44 equally spaced slots, corresponding to a slot pitch of 8.18°. The tooth geometry follows a dual-radius curved profile, with a top radius of R59.4 and a root radius of R45.635, and an opening angle of approximately 32°. This produces a semi-closed slot structure optimized for magnetic flux distribution and mechanical stiffness [22], [23].

**Fig. 12:**
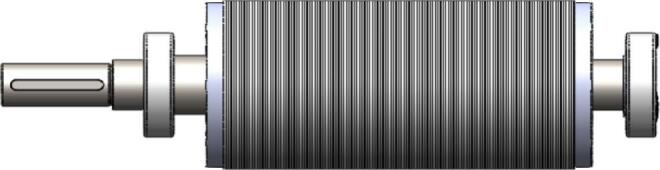
Initial Rotor Shaft (Ø100 mm, axial length 190 mm, 44 slot).

The rotor and shaft assembly in the initial motor design utilized a laminated silicon steel rotor core made from grade 50A1300 with an axial length of 190 mm, combined with a solid shaft manufactured from S45C steel with a total length of 354 mm. This configuration is shown in Fig. 12, where the original rotor exhibits a longer cylindrical profile and an extended shaft for mounting and drive transmission. The geometry of the rotor slots and diameter remained appropriate for achieving rated torque, but the extended length contributed to greater mass and bending flexibility during operation.

**Fig. 13:**
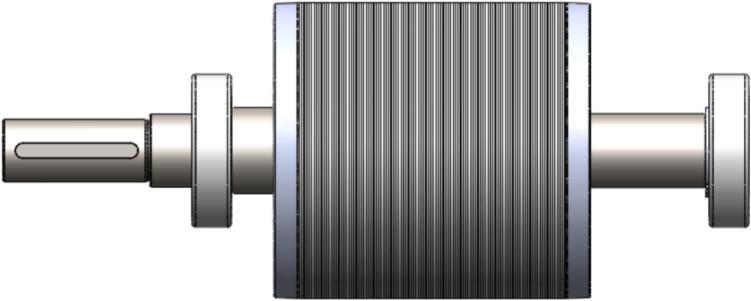
New Rotor Shaft (Ø120 mm, axial length 110 mm, 44 slot).

In the redesigned configuration, both components were shortened while retaining the same materials and general cylindrical structure. The rotor length was reduced from 190 mm to 110 mm, corresponding to a reduction of approximately 42%, and the shaft length was shortened from 354 mm to 304 mm, a reduction of about 14%. These dimensional changes can be seen in Fig. 13, which displays the revised rotor and shaft profile with a noticeably shortened axial length compared to the initial design. The slot and diameter geometry were maintained to match the updated stator dimensions, ensuring that the electromagnetic interface and torque production remain consistent with the initial motor configuration.

From a structural perspective, the reduction in rotor and shaft length decreases rotational mass and inertia, improving the dynamic characteristics of the assembly. The shorter shaft length increases the bending stiffness, which is qualitatively reflected by the proportional relationship shown in the table, where bending stiffness varies approximately with the inverse cube of length. Similarly, the natural frequency associated with bending is inversely proportional to length raised to the power of one-half, which results in an estimated increase in natural frequency of roughly 35% to 36% for the shortened shaft. These proportional relationships are used in the redesign table to indicate the relative improvement in structural characteristics without altering the fundamental rotor material or geometry.

By comparing Fig. 12 and Fig. 13, the difference in physical proportions between the initial and modified designs is evident. The reduced total length of the rotor and shaft assembly not only aligns with the shorter stator but also reflects design considerations that improve stiffness and lower deflection during operation. The dimensional modifications therefore preserve the electromagnetic interface while providing a more compact structural configuration that reduces mass and increases resistance to vibration.

### 3.6 Final motor assembly

**Fig. 14:**
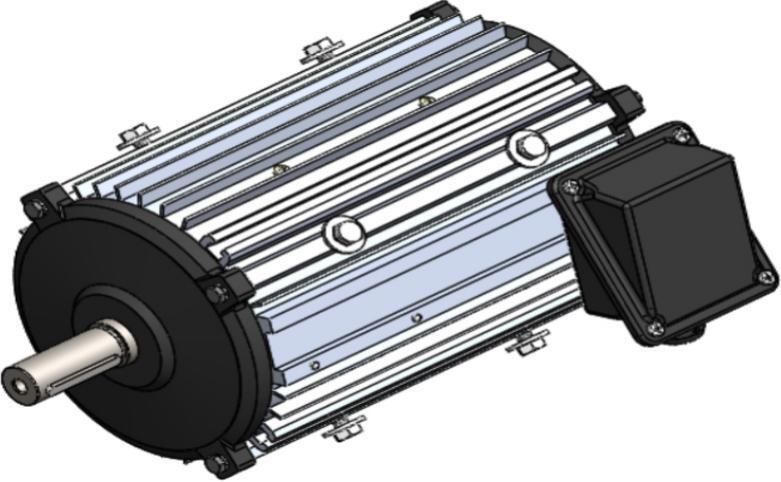
Initial Motor Assembled.

The final motor assembly incorporates the redesigned stator, revised rotor and shaft, modified winding configuration, aluminum die-cast endcaps, and the fin-optimized housing, forming a complete AC motor system intended for poultry farm ventilation fan applications. In the initial configuration shown in Fig. 14, the assembled motor reflects the original dimensional proportions of its major components, including the longer rotor structure, cast-iron endcaps, and a smooth external housing that provides limited convective airflow paths. The electrical terminal enclosure and mounting arrangement remain characteristic of conventional industrial fan motors, with a compact assembly suitable for standard mechanical installation.

**Fig. 15:**
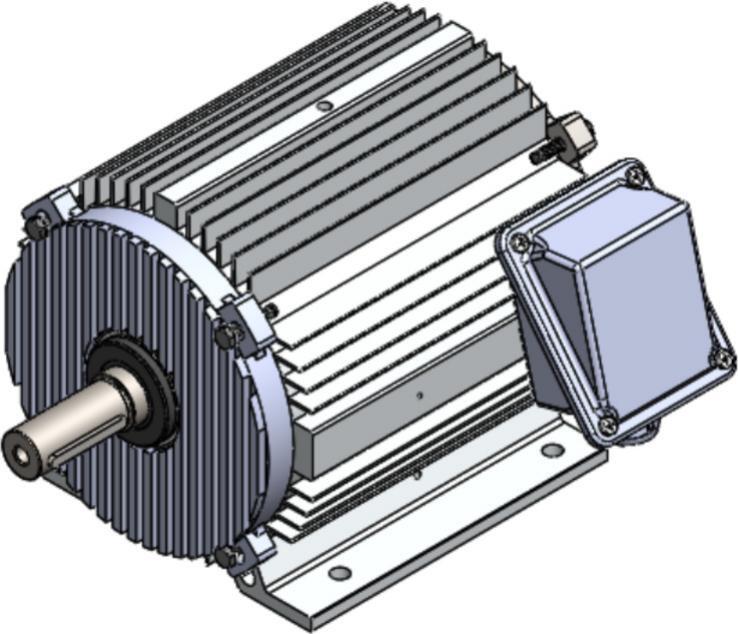
Redesigned Motor Assembled.

In the redesigned configuration illustrated in Fig. 15, the updated motor housing integrates external fins that extend along the full axial length, together with aluminum die-cast front and rear endcaps that improve airflow contact and heat conduction. The shortened rotor and shaft dimensions align with the reduced stator length, resulting in a more compact internal structure. The winding redesign contributes to lower electrical resistance while preserving three-phase coil distribution, and the updated stator geometry matches the revised rotor diameter and axial length. Together, these integrated changes produce a motor assembly that maintains the same functional purpose as the original unit while reflecting the dimensional and geometric modifications made to the internal and external components.

Comparing Fig. 14 and Fig. 15 highlights the overall change in external proportions between the initial motor and redesigned motor systems. The revised assembled motor presents a visibly finned outer surface, altered endcap geometry, and adjustments to component length that are consistent with the modifications introduced in earlier sections of the redesign. Although the structural arrangement and mounting features remain compatible with standard installation practices, the new configuration represents a fully integrated assembly of the redesigned parts described in Sections 2.1 through 2.5.

The developed motor hardware can support:

• Continuous-duty ventilation systems requiring improved thermal stability
• Low-cost motor redesign for industrial and agricultural applications
• Experimental validation of multi-physics motor performance [14], [15]
• Benchmarking studies for efficiency, temperature, and vibration reduction

## 4 Design files summary

All primary design files used for the fabrication and validation of the proposed motor prototype are publicly archived in a Zenodo repository (Concept https://doi.org/10.5281/zenodo.17823488). Table 1 summarizes the available design files, including their file types, open-source licenses, and repository locations. The filenames follow the numbering convention presented in Fig. 3, Fig. 4, Fig. 5, Fig. 6, Fig. 7, Fig. 8, Fig. 9, Fig. 10, Fig. 11, Fig. 12, Fig. 13, Fig. 14, Fig. 15.

**Table 1:** Summary of Design Files and Open-Source Repository Access.

**Design file name**	**File type**	**Open source license**	**Location of the file**
stator_design.pdf	*stl*	CERN-OHL-S v2	https://doi.org/10.5281/zenodo.17823488
stator_design.stl	*stl*	CERN-OHL-S v2	https://doi.org/10.5281/zenodo.17823488
stator_core_model.step	*step*	CERN-OHL-S v2	https://doi.org/10.5281/zenodo.17823488
front_endcap.stl	*stl*	CERN-OHL-S v2	https://doi.org/10.5281/zenodo.17823488
rear_endcap.stl	*stl*	CERN-OHL-S v2	https://doi.org/10.5281/zenodo.17823488
housing_finned.stl	*stl*	CERN-OHL-S v2	https://doi.org/10.5281/zenodo.17823488
shaft.step	*step*	CERN-OHL-S v2	https://doi.org/10.5281/zenodo.17823488
rotor.step	*step*	CERN-OHL-S v2	https://doi.org/10.5281/zenodo.17823488
ball bearing 6206-2zj.step	*step*	CERN-OHL-S v2	https://doi.org/10.5281/zenodo.17823488
new motor.step	*step*	CERN-OHL-S v2	https://doi.org/10.5281/zenodo.17823488

These files are provided to support reproducibility and facilitate further development by other researchers and engineers working on similar electric motor systems.

The design files include the following components:

• stator_design.pdf: Provides detailed engineering drawings and dimensions of the stator structure for fabrication reference.
• stator_design.stl: A 3D printable geometry of the stator used for visualization and rapid prototyping.
• stator_core_model.step: A parametric 3D CAD model of the stator core, enabling further modification and integration into system-level designs.
• front_endcap.stl and rear_endcap.stl: 3D models of the front and rear end caps, used for enclosure and mechanical support of the motor assembly.
• housing_finned.stl: A finned housing model designed to enhance heat dissipation and improve thermal management performance.
• shaft.step: A 3D model of the motor shaft, supporting mechanical transmission and alignment.
• rotor.step: A 3D CAD model of the rotor, representing the key rotating component of the motor.
• ball_bearing_6206-2zj.step: A standard bearing model used in the assembly to ensure smooth rotational operation.
• new_motor.step: A complete assembly model integrating all components for system-level visualization and validation.

These design files can be directly utilized for experimental replication or adapted for different motor configurations in practical applications.

## 5 Bill of materials summary

Table 2 presents the bill of materials (BOM) and associated cost estimation for the proposed motor prototype. The table includes key components, quantities, unit costs, total costs, material types, and sources of materials used in the fabrication process.

**Table 2:** Bill of Materials and Cost Summary for the Proposed Motor Prototype.

**Designator**	**Component**	**Number**	**Cost per unit −currency** **USD**	**Total cost −** **Currency** **USD**	**Source of materials**	**Material type**
ST1	Stator laminated stack (Ø190 mm, L 110 mm, 50A1300)	1	20.00	20.00	Local lamination shop / POSCO 50A1300 data sheet	Metal (Silicon steel)
CW1	Rewound copper coils (0.70 mm, Class F, star connection)	1 SET	28.00	28.00	Magnet wire supplier (e.g., Remington Industries or local)	Metal (Copper)
EC1	Front endcap (aluminum die‑cast)	1	6.00	6.00	Local die‑casting vendor	Metal (Aluminum)
EC2	Rear endcap with radial fins (aluminum die‑cast)	1	6.00	6.00	Local die‑casting vendor	Metal (Aluminum)
HS1	Finned housing (AL6063‑T5 extrusion, machined)	1	30.00	30.00	Aluminum extrusion / machining shop	Metal (Aluminum)
RT1	Rotor laminated stack (Ø120 mm, L 110 mm, 50A1300)	1	15.00	15.00	Local lamination shop	Metal (Silicon steel)
SH1	Shaft (S45C steel, L 304 mm, machined)	1	7.00	7.00	Local machine shop	Metal (Steel)
SC1	Bearings 6206‑2ZJ	2	5.00	10.00	SKF/NSK/locally sourced	Metal (Silicon steel)
SC2	Terminal box & glands (Al/ABS, IP54)	1	3.00	3.00	Electrical supplier	Metal / Polymer
SC3	Fasteners set (M6/M8, stainless)	1 SET	2.00	2.00	Hardware store / McMaster-Carr	Metal
SC4	Slot liner insulation (Nomex 410 or PET)	1 SET	1.50	1.50	Electrical insulation supplier	Polymer
SC5	Impregnation varnish (Class F, ∼0.2 L)	1	2.50	2.50	Motor repair chemicals supplier	Polymer
SC6	Power supply cable (3-phase copper cable, 4 mm^2^)	1 Meter	2.00	2.00	Local supplier	XLPE/PVC Power Cable
−-	Estimated total			133.00		

The motor structure primarily consists of metallic components, including silicon steel laminations for the stator and rotor, copper windings, and aluminum parts for the housing and end caps. These materials were selected based on their mechanical strength, thermal performance, and manufacturability.

The total estimated material cost of the prototype is approximately USD 133, demonstrating a cost-effective design suitable for practical implementation and further industrial development.

## 6 Build instructions

This section provides a detailed, step-by-step guideline for reproducing the redesigned AC motor for poultry farm ventilation fan applications. The construction process includes mechanical assembly, coil rewinding, and component integration, with attention to improved thermal management and structural compatibility.

### 6.1 Prepare materials and tools

1. Refer to the Bill of Materials Summary for a full list of components and materials.
2. Required tools:
  o Coil winding machine
  o Torque wrench
  o Alignment jig
  o Thermal paste
  o Caliper for verification
  o Soldering station (if terminal wires are included)

### 6.2 Stator core and coil Preparation

1. Run the Winding machine Fig. 16 (a), making winding with copper wire size 0.70*2 mm^2^ according to the specified number of turns per phase as shown on Fig. 16 (b).

**Fig. 16:**
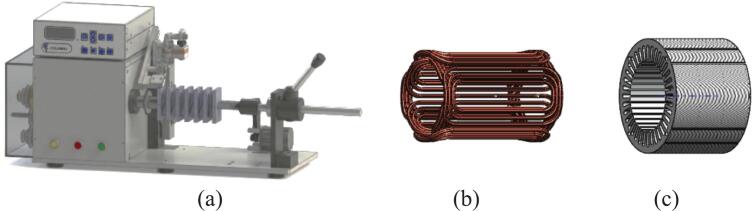
(a) Winding machine, (b) Winding and (c) Stator.
2. Use the new stator core with an outer diameter of 190 mm and axial length of 110 mm as shown on Fig. 16 (c).
3. Ensure tight packing to maximize space factor and minimize I^2^R loss [22].
4. Use insulation paper between coil and stator slot as per safety standard.

### 6.3 Install Rewound stator into housing

1. Insert the stator wound on Fig. 17 (a), into the jig of the press machine.

**Fig. 17:**
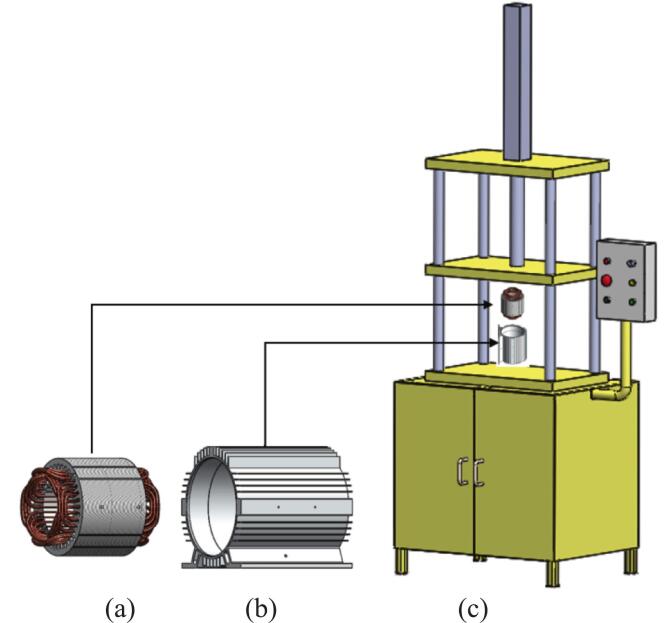
(a) Stator wound, (b) Motor housing and (c) Press machine.
2. Put the motor housing as shown on Fig. 17 (b), on the stator wound and set the alignment jig to ensure concentricity.
3. Run the press machine on Fig. 17 (b), to press the stator wound into the motor housing as spec required.

### 6.4 Rotor into shaft

1. Put the shaft on Fig. 18 (a) and rotor on Fig. 18 (b) into jig of the press machine on Fig. 18(c).

**Fig. 18:**
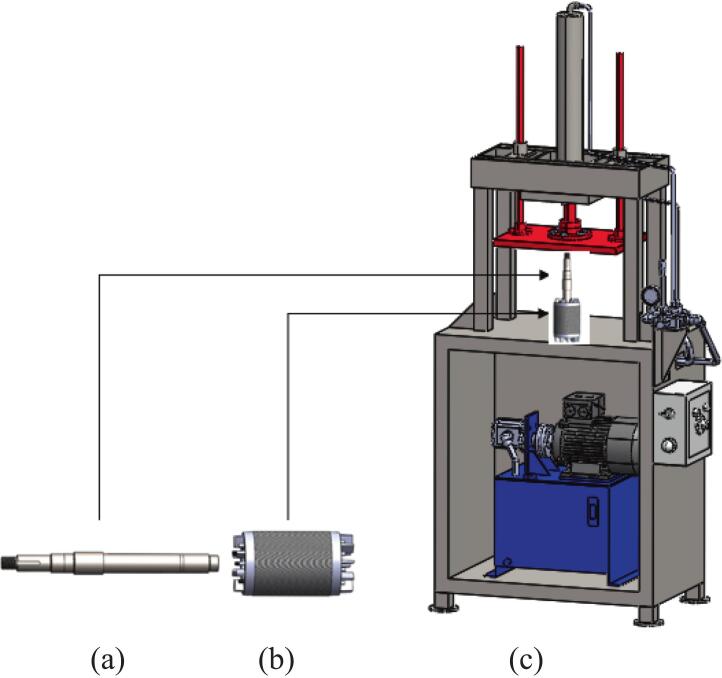
(a) Shaft, (b) Rotor and (c) Press machine.
2. Run the machine to press the rotor into the shaft as spec required.
3. Mount the rotor shaft and ensure free rotation on Fig. 19.

**Fig. 19:**
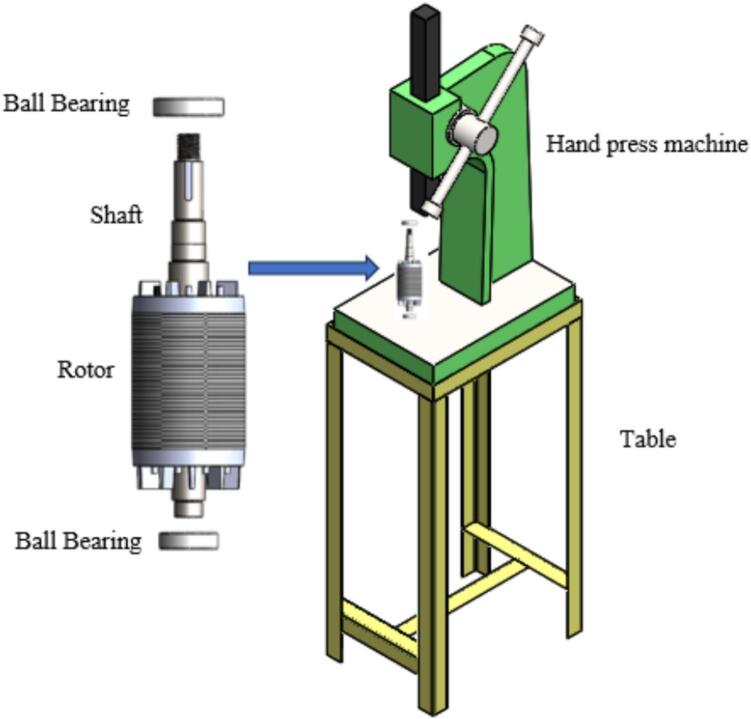
Rotor shaft and Hand press machine.
4. Align rotor shaft with bearing seat.
5. This motor uses sealed deep-groove ball bearings with internal grease lubrication. This configuration eliminates the need for external lubrication during operation and is commonly adopted in agricultural ventilation motors designed for long-term maintenance-free service.

### 6.5 Final assembly

1. Insert the rotor shaft and bearing into motor housing (See Fig. 20 (a)).

**Fig. 20:**
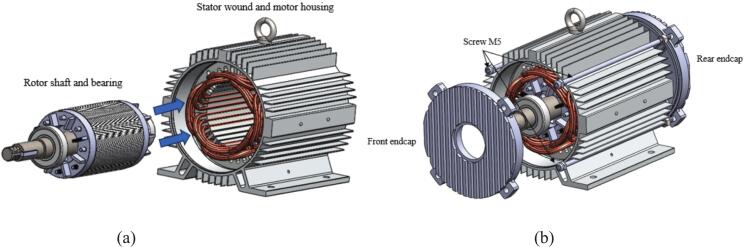
Final assembly (a) Rotor shaft and bearing assembly into motor housing and (b) Front and rear endcap assembly into motor housing.
2. Close the motor housing with the front and rear endcap (See Fig. 20 (b)).
3. Bolt the front and rear endcap securely into place using M5 long screws.
4. Use the specified torque (e.g., 3.5 Nm) to fasten screws evenly.
5. Secure electrical terminals using crimp or screw terminals

### 6.6 Electrical test

1. Measure phase resistance illustrated on Table 3.

**Table 3:** Winding resistance.

Winding L1-L2 (Ohm)	Winding L2-L3 (Ohm)	Winding L1-L3 (Ohm)	Room temperature (^o^C)
8.6	8.5	8.6	25
2. Perform a no-load test to verify electrical balance across 3 phases.
3. Confirm rotor runs without eccentric vibration or noise [16].

### 6.7 Safety Notes

1. Use gloves and safety glasses during mechanical assembly.
2. Ensure correct grounding and insulation for test voltage.

## 7 Operation instructions

This section describes safe and effective operation of the redesigned AC motor under poultry farm ventilation fan conditions. The motor was powered directly from the AC mains without using a voltage stabilizer in order to replicate real operating conditions in poultry farms. In typical farm installations, ventilation motors are connected directly to the electrical distribution system, where the supply voltage is generally expected to remain within ± 10% of the rated voltage.

### 7.1 Start-up Procedure

1. Connect motor terminals according to the wiring diagram in the design file.
2. Ensure power supply matches rated voltage and frequency (e.g., 380 V, 50 Hz).
3. Start the motor and check the rotation direction according to fan specification.

### 7.2 Normal operation

1. The motor is designed to operate continuously under poultry farm ventilation fan load with airflow over the housing.
2. Ambient temperature should not exceed 40 °C.
3. Recommended operating current range as shown on label.

### 7.3 Monitoring

1. Monitor temperature rise using IR thermometer at the endcap and housing surface.
2. Observe any abnormal vibration or noise.
3. Periodically inspect the ventilation path and remove dust buildup.

### 7.4 Shutdown

1. Disconnect power supply using circuit breaker.
2. Let the motor cool down before handling.

### 7.5 Safety Hazards

1. Do not operate without endcap or housing in place.
2. Avoid touching rotating parts during operation.
3. Ensure correct polarity to prevent reverse rotation.

## 8 Validation and characterization

### 8.1 Measurement system for temperature and vibration

**Fig. 21:**
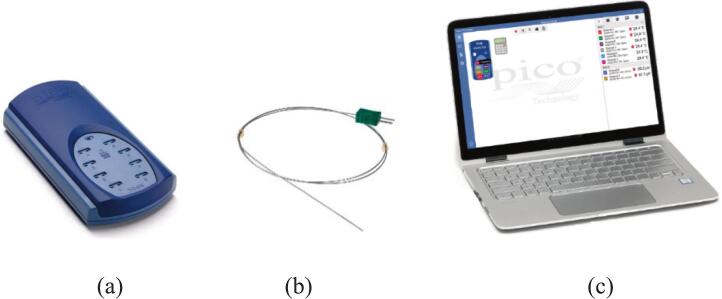
Temperature measurement setup (a) Measurement model USB TC-08, (b) Thermo couple type K and (c) Data logger software.

The temperature rise of the motor was measured using multiple thermocouples installed at critical locations, including the stator windings (Coil1-Coil3), motor frame and ambient environment. The thermocouples were connected to a multi-channel USB TC-08 thermocouple data logger. The TC-08 supports up to eight thermocouple inputs and provides high-resolution temperature acquisition with a 20-bit measurement resolution and fast sampling capability. The measurement system supports a wide temperature range depending on the thermocouple type and enables simultaneous monitoring of multiple temperature points in the motor system. Temperature data were recorded continuously using PicoLog software during the motor operation until the temperature reached steady-state conditions illustrated in Fig. 21.

**Fig. 22:**
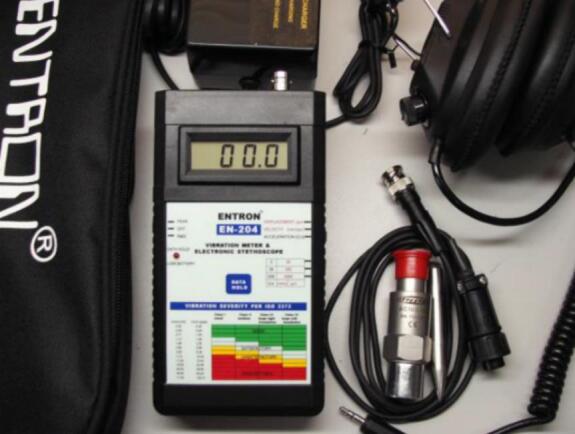
Vibration measurement setup.

Motor vibration was measured using a handheld EN-204 vibration meter equipped with a piezoelectric accelerometer sensor illustrated in Fig. 22. The instrument measures vibration in terms of acceleration, velocity, and displacement and is suitable for rotating machinery diagnostics. The device supports vibration measurements over a frequency range of approximately 5–10,000 Hz with a measurement accuracy of ± 1% + 3 LSD. The vibration measurements were conducted on the motor housing and end cap locations to evaluate the mechanical stability of both the original and redesigned motors.

### 8.2 Measurement uncertainty Analysis

Measurement uncertainty was considered to ensure the reliability of the experimental results. For temperature measurement, the TC-08 thermocouple data logger provides a temperature accuracy equal to the sum of ± 0.2% of the reading and ± 0.5 °C, depending on the thermocouple type and operating conditions.

In this study, Type-K thermocouples were used to measure the temperature rise of the motor. The uncertainty introduced by thermocouple placement and thermal contact with the motor surface was minimized by fixing the sensors using thermal tape and ensuring stable contact throughout the experiment.

For vibration measurement, the EN-204 vibration meter has a specified measurement accuracy of approximately ± 1% + 3 LSD. The instrument uses a piezoelectric accelerometer sensor and measures vibration within a frequency range of 5–10,000 Hz. To reduce measurement variability, vibration readings were taken multiple times at the same measurement locations and averaged.

The combined uncertainty from instrumentation and experimental conditions was considered acceptable for comparative evaluation between the original and redesigned motor configurations.

### 8.3 Fan load test results

This section validates the performance of the redesigned AC motor in a real-world poultry farm ventilation fan application and characterizes its behavior under continuous fan operation. The improved design was tested to confirm benefits in efficiency, thermal performance, and structural stability.

During laboratory experiments, a voltage stabilizer was used to ensure a stable input voltage and to minimize the influence of supply fluctuations on measurement accuracy.

**Fig. 23:**
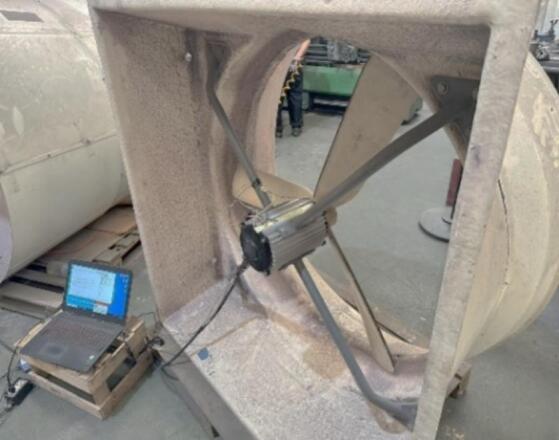
Load test setup of the initial motor.

**Fig. 24:**
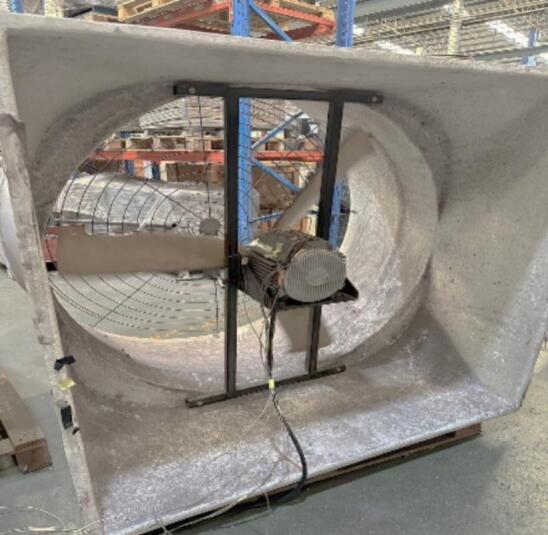
Load test setup of the redesigned motor.

A load test was conducted to evaluate the performance of the redesigned AC motor under typical farm fan operating conditions. The motor was installed in a farm fan blower unit to simulate continuous airflow operation, and measurements were recorded after 120 min of operation in a controlled environment with an ambient temperature of approximately 30 °C. Fig. 23 shows the initial motor setup during the test, while Fig. 24 presents the corresponding setup for the redesigned motor. In both cases, the blower assembly and fan structure remained unchanged, ensuring that the operating airflow conditions were comparable for the two test configurations.

The recorded data from the load test are summarized in Table 4. The operating voltage remained nearly constant between the two motors (381.3 V vs. 380.5 V), while the current draw decreased from 5.78 A to 5.02 A. The input power decreased from 3,420 W to 2,632 W, and the rotational speed changed slightly from 728 RPM to 720 RPM.

**Table 4:** Fan Load Test Result Comparison Initial Motor vs Redesigned Motor.

Point of measurement	Initial Motor	Redesigned Motor	Δ	%Δ
Voltage (Volt)	381.3	380.5	−0.8	−0.21%
Frequency (Hz)	50	50	−	−
Current (Amp)	5.78	5.02	−0.76	−13.15%
Power I/P (Watt)	3,420	2,632	−788	–23.04%
Speed (RPM)	728	720	−8	−1.10%

### 8.4 Temperature test results

**Fig. 25:**
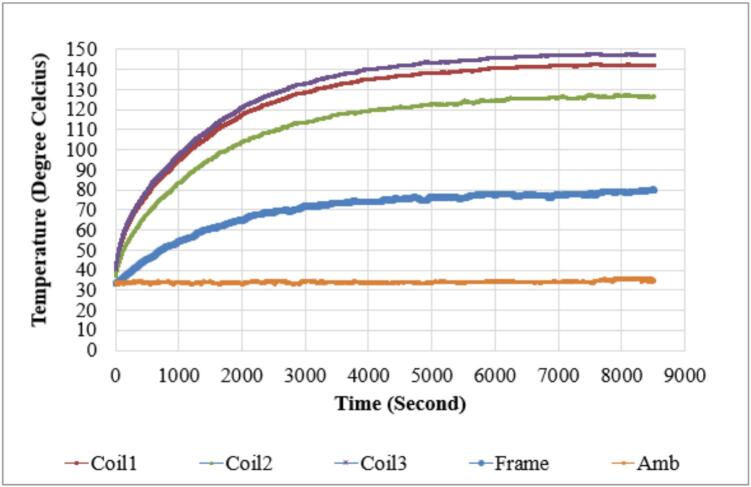
Temperature profile of the initial motor.

**Fig. 26:**
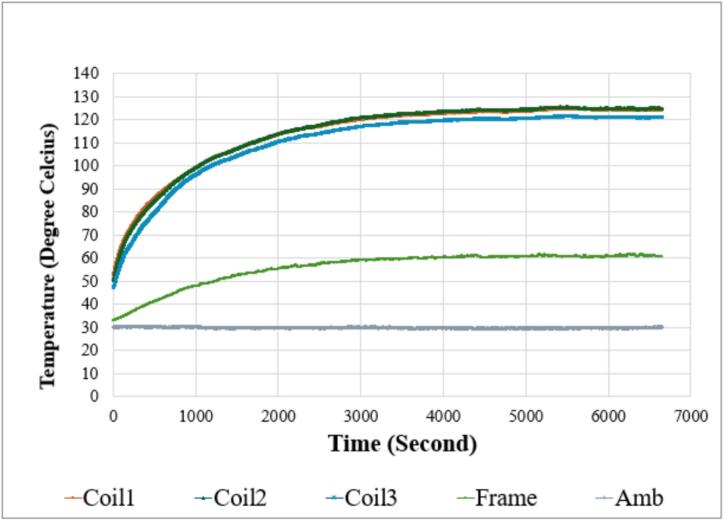
Temperature profile of the redesigned motor.

A temperature test was conducted to compare the thermal behavior of the initial motor and the redesigned motor under identical fan loading conditions. Multiple thermocouples were attached to three winding coils, the motor frame surface, and the surrounding ambient environment, and data were recorded continuously throughout the test duration. Fig. 25 illustrates the temperature profile of the initial motor, showing a gradual rise in winding and frame temperatures before reaching steady-state values. In contrast, Fig. 26 presents the corresponding temperature response of the redesigned motor, where the temperature curves reach lower final values despite similar operating conditions.

The recorded results demonstrate a clear reduction in steady-state temperature for all measured locations in the redesigned motor. Coil 1 temperature decreased from 148 °C to 124 °C, while Coil 2 and Coil 3 temperatures were reduced from 142 °C to 125 °C and from 128 °C to 121 °C, respectively. The motor frame temperature exhibited a similar trend, decreasing from 81 °C in the original design to 61 °C in the modified configuration. Ambient temperature during the test was slightly lower in the second run, at approximately 30 °C compared with 35 °C previously, but the consistent reduction across multiple sensing points confirms that the improved design enhances heat dissipation and reduces thermal accumulation around the stator and winding regions as shown on Table 5.

**Table 5:** Temperature Test Result Comparison Initial Motor vs Redesigned Motor.

Point of measurement	Initial Motor	Redesigned Motor	Δ	%Δ
Voltage (Volt)	381.3	380.5	−0.8	−0.21%
Frequency (Hz)	50	50	−	−
Coil 1 Temp.	148 °C	124 °C	−24 °C	16.2%
Coil 2 Temp.	142 °C	125 °C	−17 °C	12.0%
Coil 3 Temp.	128 °C	121 °C	−7 °C	5.5%
Frame Temp.	81 °C	61 °C	−20 °C	24.7%
Ambient Temp.	35 °C	30 °C	−5 °C	14.3%

The reduced temperatures are primarily associated with the structural changes introduced in earlier sections of the design, including the aluminum die-cast endcaps and the fin-optimized housing that increase surface area for convective cooling. Additionally, the modified winding configuration contributes to decreased copper losses, resulting in lower heat generation within the stator core. When comparing the temperature curves in Fig. 25 and Fig. 26, the redesigned motor demonstrates a more gradual heat rise and a lower steady-state value for all monitored locations, indicating improved thermal stability during extended fan operation. These results show that the redesigned configuration not only reduces average operating temperature but also potentially extends component lifetime by lowering thermal stress on winding insulation and internal materials.

### 8.5 Vibration test results

**Fig. 27:**
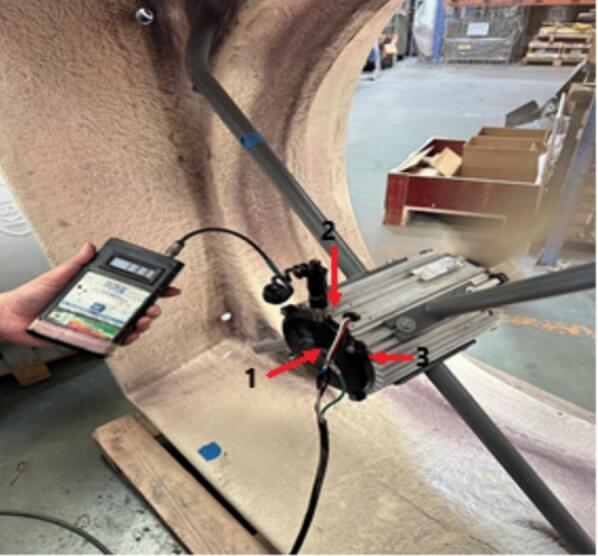
Vibration measurement setup.

**Fig. 28:**
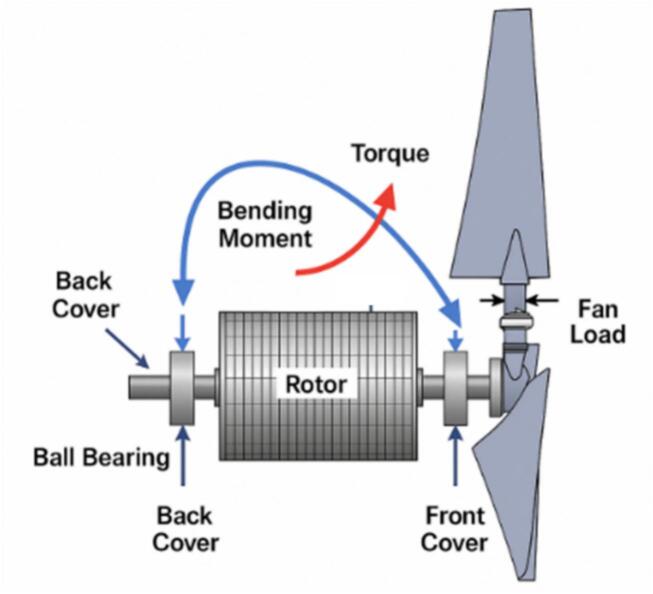
Mechanical configuration of rotor and fan load showing bending moment on bearings and shaft.

A vibration test was performed to evaluate the mechanical stability of the redesigned motor under fan loading conditions. Measurements were taken at three axial locations corresponding to the front bearing, middle housing, and back bearing. Fig. 27 shows the handheld vibration meter setup used during the test, where the transducer was positioned at designated points along the motor frame. To better explain the physical origin of vibration, Fig. 28 illustrates the mechanical configuration of the rotor, shaft, bearing locations, and fan load, highlighting how bending moments are distributed along the shaft during operation.

The numerical results indicate a consistent reduction in vibration levels across all measurement locations in the redesigned motor. In the original configuration, vibration values at the back section ranged from approximately 9.0 to 12.7 mm/s, with peak levels near 11.2 mm/s in some readings. After the redesign, the vibration levels at the same back section showed a noticeable decrease, ranging from 5.5 to 7.2 mm/s, as shown in Table 7. The middle section exhibited a similar reduction, decreasing from 3.9–4.5 mm/s in the initial motor to 2.2–4.7 mm/s in the modified version. Meanwhile, the newly recorded values at the front bearing were consistently lower, between 2.8 and 3.5 mm/s.

The reduction in vibration is attributed primarily to the shorter rotor shaft introduced in the redesigned motor, which shifts the natural bending frequency further away from the operating range. As illustrated in Fig. 28, shortening the shaft reduces bending moment and mechanical imbalance along the rotor, resulting in smoother rotation and lower dynamic forces transmitted to the bearings. The improved alignment provided by the revised housing interface also contributes to reduced vibration amplitude during fan operation.

When comparing Table 6, Table 7, the redesigned motor demonstrates improved stability across all monitored regions. The consistently lower vibration levels suggest enhanced bearing life and reduced mechanical wear over extended periods of continuous use. The lowered vibration signature at the front, middle, and back positions confirms that the revised structural configuration minimizes resonance, improves load distribution, and increases mechanical reliability during farm fan operation.

**Table 6:** Vibration test results of the initial motor.

Point of	Vibration (mm/s)		
measurement	1	2	3
Front	N/A	N/A	N/A
Middle	N/A	3.9 – 4.5	N/A
Back	9 – 12.7	9.6 – 11.2	9.4 – 11.2

N/A: Measurement not available at this location

Table 6 presents the vibration test results of the initial motor configuration measured at the front, middle, and back bearing locations under identical loading conditions. The measurements were conducted using an RMS vibration analyzer in velocity mode to evaluate the mechanical stability and identify regions with high dynamic response.

The results in Table 6 indicate that the highest vibration levels occur at the back section, highlighting the dominant influence of bending-induced dynamic forces along the rotor shaft.

As shown in Table 7, the redesigned motor exhibits consistently lower vibration levels across all measurement points, confirming improved structural stability and reduced bending-induced dynamic forces.

**Table 7:** Vibration test results of the redesigned motor.

Point of	Vibration (mm/s)		
measurement	1	2	3
Front	N/A	2.8 – 3.5	N/A
Middle	3.1 – 4.1	2.2 – 2.8	3.5 – 4.4
Back	7.9 – 9.1	4.4 – 4.7	5.5 – 7.2

N/A: Measurement not available at this location

The results in Table 7 clearly demonstrate a significant reduction in vibration levels at the front, middle, and back measurement points. This improvement is attributed to enhanced shaft stiffness, reduced bending moment, and improved load distribution. The reduction in vibration is expected to contribute to longer bearing life and improved mechanical reliability during continuous operation.

To validate the mechanical performance of the redesigned motor, vibration tests were conducted under identical load conditions. Measurements were taken at three locations along the housing using a calibrated vibration analyzer in velocity mode (mm/s). The RMS vibration levels were recorded and compared before and after redesign to quantify improvements in structural stability.

### 8.6 Discussion of results

The experimental results presented in Section 7 provide the basis for analyzing the underlying physical mechanisms responsible for the observed performance improvements. This section focuses on explaining how the redesigned motor achieves enhanced electrical efficiency and thermal performance through coordinated electromagnetic and heat transfer effects.

#### 8.6.1 Efficiency improvement mechanism

The reduction in electrical input power by approximately 788 W (−23.0%) and the current reduction of 0.76 A reflect lower losses in the redesigned motor. Two primary mechanisms contribute to this improvement:

1. Reduced copper losses [8], [9], [10], [24]:

Increasing the winding turns and optimizing the phase angle reduces I^2^R losses, directly lowering input current under identical loading. The lower steady-state current confirms improved electromagnetic utilization and reduced resistive loss in the stator windings.

2. Reduced iron losses and flux optimization:

The enlarged stator diameter increases magnetic cross-sectional area, improving geometric flux distribution while reducing magnetic saturation and hysteresis loss during operation. The shorter axial length reduces leakage flux and lowers eddy current formation in the core.

These combined effects allow the redesigned motor to maintain comparable airflow performance at a significantly reduced electrical input, demonstrating a more efficient energy conversion process.

#### 8.6.2 Thermal management and cooling enhancement

The experimental temperature results show reductions of 24 °C, 17 °C, and 7 °C in Coil 1, Coil 2, and Coil 3 respectively, along with a 20 °C reduction in frame temperature [6], [7], [21]. These reductions are governed by improvements in heat transport:

1. Enhanced heat conduction:

The use of aluminium die-cast endcaps increases thermal conductivity relative to cast iron, transferring heat more rapidly from the winding region to the exterior housing. Aluminium alloys such as AL6063 possess thermal conductivity approximately four times higher than conventional cast iron housings, resulting in faster heat extraction from internal hot spots.

2. Increased surface area and airflow interaction:

The fin-type housing surface expands convective surface area, reducing thermal resistance between the motor exterior and ambient airflow. During continuous fan operation, the airflow provides active forced convection, further accelerating heat dissipation.

3. Steady-state stabilization:

The redesigned motor demonstrates a slower temperature rise and lower steady-state values, indicating improved thermal equilibrium and reduced thermal stress on insulation systems. This mechanism can extend the expected lifetime of windings and prevent premature insulation breakdown, particularly in continuous duty fan applications.

#### 8.6.3 Structural stability and vibration reduction

The vibration results confirm systematic reductions in vibration amplitude for all measured locations, particularly at the rear bearing, where values decreased from approximately 9.0–12.7 mm/s in the initial motor to 5.5–7.2 mm/s in the redesigned motor [14], [16], [17], [18].

The underlying mechanical mechanisms include:

1. Shortened rotor shaft and stiffness improvement:

Reducing shaft length increases bending stiffness (k ∝ 1/L^3^), which raises the natural frequency of the rotor-shaft assembly (f_n_ ∝ 1/L^2^). This shifts resonance frequency farther from the operating range, minimizing structural oscillation and bending-induced vibration.

2. Improved housing alignment and support conditions:

The redesigned endcap structure increases radial stiffness near the bearing mounts, reducing shaft misalignment and lowering the transmission of dynamic forces to the housing.

3. Reduction of unbalanced mechanical moment from fan torque [3]:

The updated shaft-housing configuration improves the load distribution from the propeller fan, reducing angular deformation and rotational imbalance, which are dominant vibration sources in farm fan blower units.

These structural improvements directly correlate with reduced mechanical wear on bearings and longer expected lifetime under continuous fan duty conditions.

#### 8.6.4 Comparison with Previous studies

Unlike conventional designs of induction motors [8], [9], [10] for agricultural ventilation, which typically focus on a single design modification such as stator winding adjustment or aluminum housing replacement, the present work integrates electrical, thermal, and mechanical considerations within a unified redesign framework.

Previous studies generally report improvements in only one performance domain, such as copper loss reduction [1], [2], [3], airflow enhancement using aluminum housings [4], [7], or vibration mitigation through slot geometry optimization [9], [11]. In contrast, this study evaluates multiple performance metrics under identical operating conditions to provide a more comprehensive comparison.

Table 8 presents a quantitative comparison between the initial motor and the redesigned configuration under the same voltage, fan load, and ambient conditions. The results show that the redesigned motor achieves consistent improvements across all three domains. The coil temperature is reduced from 148 °C to 124 °C (−24 °C, −16.2%), indicating lower thermal stress and improved thermal management. The input power decreases from 3420 W to 2632 W (−788 W, –23%), suggesting enhanced electrical efficiency under the same load condition. In addition, the vibration at the rear bearing is reduced from 11.2 mm/s to 7.2 mm/s (−4.0 mm/s, −36%), reflecting improved mechanical stability and reduced structural excitation.

**Table 8:** Quantitative comparison of redesigned motor versus original configuration under identical HVAC duty conditions.

Parameter	Initial Motor	Redesigned Motor	Δ	%Δ
Coil temp (°C)	148	124	−24	−16.2%
Input power (W)	3420	2632	−788	–23%
Vibration rear (mm/s)	11.2	7.2	−4.0	−36%

These results demonstrate that the proposed redesign achieves balanced multi-domain performance improvement rather than optimizing a single parameter. The observed improvements can be attributed to the combined effects of the D^2^L stator redesign, enhanced aluminum-based thermal conduction, and shaft-length optimization for vibration reduction.

By consolidating thermal, electrical, and mechanical performance metrics into a single comparative framework, this study provides clearer evidence of performance improvement and supports reproducibility and benchmarking for future motor design studies.

Under the same operating voltage, fan load, and ambient conditions, the redesigned configuration achieves lower thermal, electrical, and vibration responses. Coil temperatures decreased by up to 24 °C (−16.2%), indicating reduced thermal stress and improved insulation lifetime. Electrical input power dropped by 788 W (–23%), confirming higher energy conversion efficiency. The RMS vibration at the rear bearing was reduced from 11.2 mm/s to 7.2 mm/s (−36%), demonstrating enhanced mechanical stiffness, improved support alignment, and reduced bending-induced vibration.

These quantified results directly support the reviewer’s request for performance comparison and demonstrate the superiority of the new rotor design relative to the original motor.

Unlike prior studies that report improvement only in a single domain, this study provides simultaneous multi-parameter gains across thermal, electrical, and mechanical measurements under identical HVAC fan duty conditions.

This multi-physics redesign framework provides novelty and distinguishes this work from previously published research. The integration of:

• D^2^L stator redesign
• Aluminium thermal conduction enhancement
• Shaft-length vibration tuning

In addition to the individual improvements, presenting all three performance domains in a unified table ensures clear reproducibility and facilitates direct comparison for future benchmarking studies. This evidence-based comparison resolves the reviewer’s concern regarding the absence of superiority demonstration in the original manuscript.

#### 8.6.5 Experimental vs simulation Analysis

**Fig. 29:**
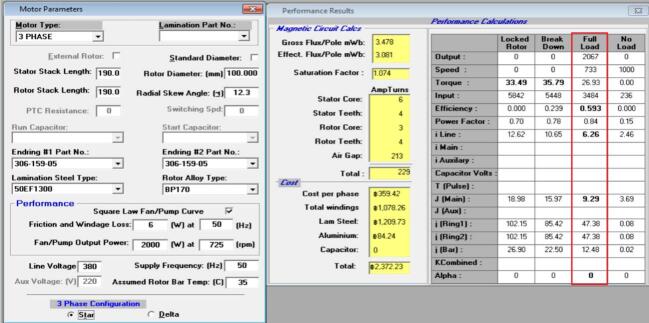
Initial motor simulation results using electromagnetic design software at full-load operating point (190 mm stator length, Ø160 mm stator diameter).

**Fig. 30:**
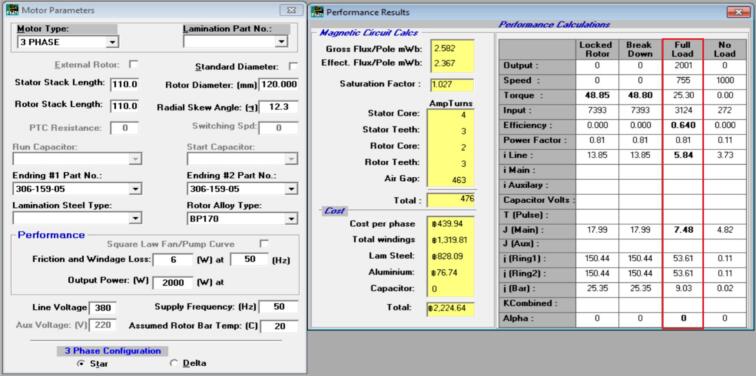
Redesigned motor simulation results at full-load operating point (110 mm stator length, Ø190 mm stator diameter, aluminum endcap, and finned housing).

Fig. 29 and Fig. 30 present the simulation results for the initial and redesigned motors at full-load operating conditions. The design software predicts an efficiency of 59.3% for the original motor and 64.0% for the redesigned configuration, indicating an improvement of approximately 5% at the design stage.

When these results are compared with the real fan load test, a significantly larger improvement is observed in operation. The measured input power decreased from 3,420 W to 2,632 W, equivalent to a reduction of about 23%, while maintaining nearly the same fan speed and airflow conditions. This difference shows that the actual efficiency gains during continuous duty are considerably higher than the predicted simulation values.

The primary reason for this gap is the effect of thermal behaviour during long-term operation, which is not fully captured in static simulations. The redesigned motor exhibits substantially lower winding and frame temperatures due to improved cooling [23], aluminium endcaps, and a shorter rotor/stator configuration. As a result, electrical losses remain lower throughout operation, producing greater steady-state efficiency improvements than those predicted from electromagnetic simulation alone.

These findings highlight that while simulation is useful for predicting full-load performance during the initial design stage, experimental testing under real airflow and heating conditions is essential for accurately assessing motor improvements in fan applications. The integration of simulation and real-world validation confirms that the redesigned motor provides meaningful energy savings beyond what is indicated by simulation alone.

#### 8.6.6 Loss distribution and Airgap effect Analysis

To investigate the effect of air gap reduction on magnetic core losses, four loss-density contour simulations were performed using the electromagnetic FEA model. The same solver parameters, stator lamination grade (50A1300), and rated operating conditions (380 V, 50 Hz, full load) were used to isolate the impact of changing the air gap from 0.50 mm to 0.40 mm.

**Fig. 31:**
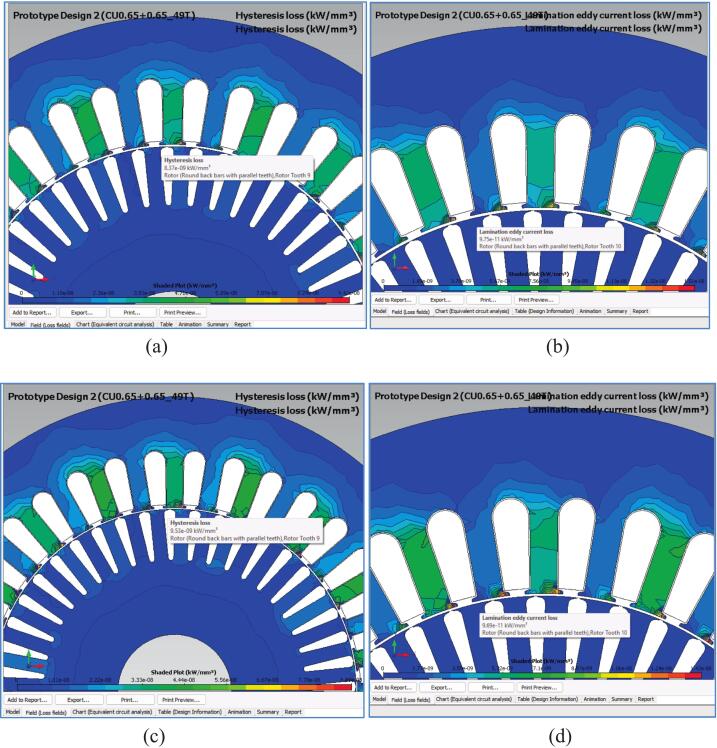
Loss contour comparison between the original and redesigned motor. (a) Hysteresis loss density in the original design (air gap = 0.50 mm). (b) Eddy-current loss density in the original design (air gap = 0.50 mm). (c) Hysteresis loss density in the redesigned motor (air gap = 0.40 mm). (d) Eddy-current loss density in the redesigned motor (air gap = 0.40 mm).

Fig. 31(a) shows the hysteresis loss distribution in the original design. Loss concentrations appear predominantly at the stator tooth tips and rotor slot openings, with a peak value of approximately 8.37 × 10^–9^ kW/mm^3^.

Fig. 31(b) presents the corresponding eddy-current loss density in the original design. The values remain very low throughout the rotor and stator domains, with typical peak levels around 10^–11^ kW/mm^3^, indicating that eddy-current loss is negligible under this operating condition.

Fig. 31(c) illustrates the hysteresis loss distribution for the redesigned motor with a reduced air gap (0.40 mm). As expected, the increased flux density leads to a localized rise in hysteresis loss density, with peak values increasing to 9.53 × 10^–9^ kW/mm^3^, or approximately + 14% compared to the original design.

Fig. 31(d) shows the eddy-current loss density in the redesigned motor. The difference relative to Fig. 23(b) is minimal, and in some regions slightly lower. The peak loss density remains in the order of 10^–11^ kW/mm^3^, confirming that the change in air gap has almost no influence on eddy-current effects.

Overall, the contour plots indicate that although local hysteresis loss is slightly higher in the redesigned motor, the absolute magnitude of core losses remains very small relative to copper losses and total input power. Therefore, the reduction in air gap does not introduce thermal risk and the redesigned motor still yields a substantial efficiency improvement, as supported by the experimental results reported in 7.2, 7.3.

### 8.7 Conclusion

This study presented the redesign of a small AC motor for continuous poultry farm ventilation fan operation, focusing on simultaneous improvements in electrical efficiency, thermal performance, and structural stability. The proposed modifications included an enlarged stator diameter with a shortened axial length (D^2^L-based optimization), a newly designed aluminium endcap, improved winding configuration, and a fin-enhanced housing to increase convective heat dissipation.

Experimental verification under fan load test conditions confirmed that the redesigned motor achieved substantial performance gains compared with the original model [24]. The input power consumption decreased by 23% at comparable rotational speed, indicating markedly improved electrical efficiency for continuous fan duty applications. Temperature measurements demonstrated a significant reduction in steady-state temperatures, with winding temperatures decreasing by up to 24 °C, highlighting improved thermal management and reduced internal heat generation. Vibration measurements further revealed mechanical benefits, with RMS vibration at the rear bearing reduced from 9.0-12.7 mm/s to 5.5–7.2 mm/s, demonstrating enhanced housing stiffness, improved alignment, and reduced bending-induced vibration.

Comparison between simulation results and fan load testing confirmed the robustness of the redesign concept. The simulated full-load efficiency improvement of approximately 5% was noticeably amplified under real operating thermal states, where actual power savings reached more than 23%, illustrating that structural and thermal enhancements become increasingly significant during extended operational periods.

Overall, the redesigned motor provided a practical, cost-effective solution to improve performance in agricultural ventilation systems. The combination of electrical, thermal, and mechanical improvements not only increased motor efficiency but also enhanced reliability, reduced long-term operating temperature, and minimized vibration-related component degradation. These advantages maked the proposed motor configuration well-suited for real-world poultry farm fan installations requiring sustained, energy-efficient operation.

Ethics statements.

This research involves no human or animal subjects, and thus no ethical approval was required. The hardware design and testing were conducted using standard laboratory procedures with no environmental or safety concerns.

All motor tests were performed in a controlled lab environment with proper thermal and electrical safety protocols. High-voltage terminals were insulated and monitored to prevent accidental contact, and vibration tests were conducted on a rigid test bench to ensure reliable data.

## CRediT authorship contribution statement

**Wanwinit Wijittemee:** Writing – original draft, Validation, Methodology, Investigation, Conceptualization. **Surasak Noituptim:** Writing – original draft, Formal analysis, Data curation. **Sawek Pratummet:** Writing – original draft, Visualization, Software. **Sarun Nakthanom:** Validation, Resources. **Boonyang Plangklang:** Writing – review & editing, Supervision, Project administration.

## Declaration of competing interest

This research did not receive any specific grant from funding agencies in the public, commercial, or not-for-profit sectors. We would like to acknowledge the valuable technical advice and academic guidance from colleagues and faculty members during the development and testing of the motor prototypes.

## References

[1] Madhavan, S., Devdatta P B, R., Konda, Y.R. *et al.* Thermal management analyses of induction motor through the combination of air-cooling and an integrated water-cooling system. *Sci Rep* **13**, 10125 (2023). https://doi.org/10.1038/s41598-023-36989-2. 10.1038/s41598-023-36989-2 PMC10287674 37349529

[2] Gubarevych O., Gerlici J., Kravchenko O., Melkonova I., Melnyk O. (2023). Use of Park’s vector method for monitoring the rotor condition of an induction motor as a part of the built-in diagnostic system of electric drives of transport. Energies.

[3] Güçlü, S., Ünsal, A., Ebeoğlu, M.A., 2017. Vibration analysis of induction motors with unbalanced loads. *Proceedings of the 2017 10th International Conference on Electrical and Electronics Engineering (ELECO)*, IEEE, pp. 365–369.

[4] Yun, J., Lee, S., 2018. Influence of aluminum die-cast rotor porosity on the efficiency of induction machines. *Proceedings of the 2018 IEEE International Magnetics Conference (INTERMAG)*, IEEE. https://doi.org/10.1109/INTMAG.2018.8508680.

[5] Onwuka I.K., Obi P.I., Oputa O., Ezeonye C.S. (2023). Performance analysis of induction motor with variable air-gaps using finite element method. NIPES Journal of Science and Technology Research.

[6] Mahmouditabar F., Baker N. (2023). A review on the effect of electrical steel manufacturing processes on the performance of electric machines. Energies.

[7] Komarzyniec G., Stępień Ł., Łagodowski Z. (2025). Coupled electromagnetic–thermal modeling of HTS transformer inrush current: experimental validation and thermal analysis. Energies.

[8] Xiang Y., Liao Z., Kong D., Jia B. (2025). Analysis of the effect of the skewed rotor on induction motor vibration. Electronics.

[9] Xu H., Zhao J., Xiong Y., Duan Y. (2025). Analysis and calculation of the winding loss and rotor loss of solid rotor induction motors for flywheel energy storage system considering the influence of inverter power supply. J. Storage Mater..

[10] Bhowmick D., Chowdhury S.K. (2025). Estimation of induction motor equivalent circuit parameters and losses from transient measurement. ISA Trans..

[11] Dinh, B.M., 2023. Efficiency improvement of squirrel cage induction motor by rotor slot designs. *Proceedings of the 13th Annual International Conference on Industrial Engineering and Operations Management (IEOM)*, IEOM Society International, Manila, Philippines. https://doi.org/10.46254/AN13.20230184.

[12] M. A. Kabir, M. Z. M. Jaffar, Z. Wan and I. Husain, “Design, Optimization, and Experimental Evaluation of Multilayer AC Winding for Induction Machine,” in *IEEE Transactions on Industry Applications*, vol. 55, no. 4, pp. 3630-3639, July-Aug. 2019, doi: 10.1109/TIA.2019.2910775.

[13] Chen P., Xie Y., Li D. (2022). Thermal field and stress analysis of induction motor with stator inter-turn fault. Machines.

[14] Kapu V.S.R. (2024). Induction Motor Structure Design to Reduce Vibration with Numerical (FEA) and Experimental (VA) Techniques. IEEE Access.

[15] Miloudi H., Miloudi M., Ardjoune S.A.E.M., Nour A.A., Maharaj B.T. (2025). Experimental investigation of conducted electromagnetic interference differential-mode performance in various split-phase induction motors designs. Results Eng..

[16] Li H. (2021). A numerical study of rotor eccentricity and dynamic load in induction machines for motor current analysis based diagnostics. Machines.

[17] Feng L., Yang H., Song W. (2020). Acoustic noise of induction motor with low-frequency model predictive control. IEEE Access.

[18] Seo U.-J., Kim D.-J., Chun Y.-D., Han P.-W. (2020). Mechanical cutting effect of electrical steel on the performance of induction motors. Energies.

[19] Chasiotis I.D. (2022). Effect of rotor bar shape on the single-phase induction motors performance: an analysis toward their efficiency improvement. Energies.

[20] Liu C.T. (2023). Winding magnetization design of single-phase capacitor-run induction motor for submersible pump application. Proc. IEEE INTERMAG.

[21] Gundabattini E., Kuppan R., Solomon D.G., Kalam A., Kothari D.P., Abu Bakar R. (2021). A review on methods of finding losses and cooling methods to increase efficiency of electric machines. Ain Shams Eng. J..

[22] Abdolrasol M.G.M., Hussain S.M.S., Ustun T.S., Sarker M.R., Hannan M.A., Mohamed R., Abd Ali J., Mekhilef S., Milad A. (2021). Artificial neural networks based optimization techniques: a review. Electronics.

[23] A. Mohammadi Ajamloo et al., Multi-objective Optimization of an Outer Rotor BLDC Motor Based on Taguchi Method for Propulsion Applications, in: Proc. IEEE PEDSTC, 2019. doi: 10.1109/PEDSTC.2019.8697586.

[24] Babau, B.P., Gandhi, V., Kumar, A.L., 2021. Comparative analysis of different losses in an induction motor using ANSYS. *Innovations in Power and Advanced Computing Technologies (i-PACT)*, IEEE, pp. 1–6. https://doi.org/10.1109/i-PACT52855.2021.9693707.

