# Supplementary material for: Thermal-stable rotor with enhanced cooling design for efficient electric motor applications
Source: HardwareX. 2026 Apr 7;26:e00770. doi: 10.1016/j.ohx.2026.e00770 (PMC13092767; doi:10.1016/j.ohx.2026.e00770)
Supplement: Supplementary Data 1 [file mmc1.docx]

**Bill of materials summary**

| **Designator** | **Component** | **Number** | **Cost per unit -currency**  **USD** | **Total cost -**  **Currency**  **USD** | **Source of materials** | **Material type** |
| --- | --- | --- | --- | --- | --- | --- |
| *ST1* | *Stator laminated stack (Ø190 mm, L 110 mm, 50A1300)* | *1* | *20.00* | *20.00* | *Local lamination shop / POSCO 50A1300 data sheet* | *Metal*  *(Silicon steel)* |
| *CW1* | *Rewound copper coils (0.70 mm, Class F, star connection)* | *1 SET* | *28.00* | *28.00* | *Magnet wire supplier (e.g., Remington Industries or local)* | *Metal (Copper)* |
| *EC1* | *Front endcap (aluminum die‑cast)* | *1* | *6.00* | *6.00* | *Local die‑casting vendor* | *Metal*  *(Aluminum)* |
| *EC2* | *Rear endcap with radial fins (aluminum die‑cast)* | *1* | *6.00* | *6.00* | *Local die‑casting vendor* | *Metal*  *(Aluminum)* |
| *HS1* | *Finned housing (AL6063‑T5 extrusion, machined)* | *1* | *30.00* | *30.00* | *Aluminum extrusion / machining shop* | *Metal*  *(Aluminum)* |
| *RT1* | *Rotor laminated stack (Ø120 mm, L 110 mm, 50A1300)* | *1* | *15.00* | *15.00* | *Local lamination shop* | *Metal*  *(Silicon steel)* |
| *SH1* | *Shaft (S45C steel, L 304 mm, machined)* | *1* | *7.00* | *7.00* | *Local machine shop* | *Metal*  *(Steel)* |
| *SC1* | *Bearings 6205‑2RS* | *2* | *5.00* | *10.00* | *SKF/NSK/locally sourced* | *Metal*  *(Silicon steel)* |
| *SC2* | *Terminal box & glands (Al/ABS, IP54)* | *1* | *3.00* | *3.00* | *Electrical supplier* | *Metal / Polymer* |
| *SC3* | *Fasteners set (M6/M8, stainless)* | *1 SET* | *2.00* | *2.00* | *Hardware store / McMaster-Carr* | *Metal* |
| *SC4* | *Slot liner insulation (Nomex 410 or PET)* | *1 SET* | *1.50* | *1.50* | *Electrical insulation supplier* | *Polymer* |
| *SC6* | *Power supply cable (3-phase copper cable, 4 mm²)* | *1 Meter* | *2.00* | *2.00* | *Local supplier* | *‎XLPE/PVC Power Cable* |
|  |  |  |  |  |  |  |
| *--* | *Estimated total* |  |  | *133.00* |  |  |
